# Perception and attitude of healthcare workers in Saudi Arabia with regard to Covid-19 pandemic and potential associated predictors

**DOI:** 10.1186/s12879-020-05443-3

**Published:** 2020-09-29

**Authors:** Mostafa A. Abolfotouh, Adel F. Almutairi, Ala’a A. BaniMustafa, Mohamed A. Hussein

**Affiliations:** 1 King Abdullah International Medical Research Center, Ministry of National Guard-Health Affairs, POB 22490, Riyadh, 11426 Saudi Arabia; 2 King Saud bin-Abdulaziz University for Health Sciences, Ministry of National Guard-Health Affairs, POB 22490, Riyadh, 11426 Saudi Arabia; 3 King Abdulaziz Medical City, Ministry of National Guard-Health Affairs, POB 22490, Riyadh, 11426 Saudi Arabia

**Keywords:** Worries, Concern, Attitude, Perception, Healthcare worker, MERS-CoV, Pandemic, Outbreak, Saudi Arabia

## Abstract

**Background:**

Healthcare workers (HCWs) face considerable mental and physical stress caring for patients with Covid-19. They are at higher risk of acquiring and transmitting this virus. This study aims to assess perception and attitude of HCWs in Saudi Arabia with regard to Covid-19, and to identify potential associated predictors.

**Methods:**

In a cross-sectional study, HCWs at three tertiary hospitals in Saudi Arabia were surveyed via email with an anonymous link, by a concern scale about Covid-19 pandemic during 15–30 April, 2020. Concerns of disease severity, governmental efforts to contain it and disease outcomes were assessed using 32 concern statements in five distinct domains. Multiple regression analysis was used to identify predictors of high concern scores.

**Results:**

A total of 844 HCW responded to the survey. Their average age was 40.4 ± 9.5 years, 40.3% were nurses, 58.2% had direct patient contact, and 77.3% were living with others. The majority of participants (72.1%) had overall concern scores of 55 or less out of a maximum score of 96 points, with an overall mean score of 48.5 ± 12.8 reflecting moderate level of concern. Three-fourth of respondents felt at risk of contracting Covid-19 infection at work, 69.1% felt threatened if a colleague contracted Covid-19, 69.9% felt obliged to care for patients infected with Covid-19 while 27.7% did not feel safe at work using the standard precautions available. Nearly all HCWs believed that the government should isolate patients with Covid-19 in specialized hospitals (92.9%), agreed with travel restriction to and/or from areas affected by Covid-19 (94.7%) and felt safe the government implemented curfew and movement restriction periods (93.6%). Predictors of high concern scores were; HCWs of Saudi nationality (*p* < 0.001), younger age (*p* = 0.003), undergraduate education (*p* = 0.044), living with others (*p* = 0.003) working in the western region (*p* = 0.003) and direct contact with patients (*p* = 0.018).

**Conclusions:**

This study highlights the high concern among HCWs about Covid-19 and identifies the predictors of those with highest concern levels. To minimize the potential negative impact of those concerns on the performance of HCWs during pandemics, measures are necessary to enhance their protection and to minimize the psychological effect of the perceived risk of infection.

## Background

In December 2019, a cluster of patients with pneumonia was linked to a seafood wholesale market in Wuhan, China, which lead to the discovery of a new betacoronavirus [1], on 7 January, 2020, named Severe Acute Respiratory Syndrome Coronavirus-2 (SARS-CoV-2) [2] that causes coronavirus disease 2019 (COVID-19). With its novelty and rapid national and international spread on 30 Jan 2020, the World Health Organization (WHO) International Health Regulation (IHR) emergency committee declared the disease a Public Health Emergency of International Concern (PHEIC). It was declared by WHO [3] as a worldwide pandemic on 11 March 2020. At the time of this writing, it has infected 7,459,741 individuals, with 3,778,537 recoveries and 419,041 deaths, with an overall estimated case fatality rate (CFR) of 5.6% [4]. On the 2nd of March 2020, a Saudi citizen coming from Iran through Bahrain was tested positive for Covid-19 and reported by the Ministry of Health as the first case in Saudi Arabia [5]. As of 11th June, 2020, Saudi Arabia had 112,288 infected cases, with 77,954 recoveries and 819 deaths [6].

Health care workers face considerable mental and physical stress caring for patients with Covid-19. Several reports around the world suggest that this stress has led some physicians to take their own life [7, 8]. Furthermore, others were overstressed and died from exhaustion [9–12]. One approach to minimize such stresses during pandemics is for hospitals to organize physician shifts with mandatory rest and meal breaks. Professional societies can also play a significant role by offering online networking to keep doctors connected to provide some level of social support. The government can also play a role by improving the benefits for HCWs and their families [13]. These initiatives can be further enhanced by understanding the level of concerns and worries among healthcare workers and provide targeted strategies that address those concerns. Along this line, several studies have investigated the self-satisfaction of HCW and their personal feelings across several important domains [14–18]. These domains cover concerns around risks posed to family members, perception of risk at the work place, and perception of the response of government to the epidemic management [14].. Understanding the concern level across these different domains can be of importance to targeted mitigation strategies.

In Saudi Arabia, a previous study has shown that HCWs had, in general, a negative attitude toward MERS-CoV infection [16]. In this study, the majority of the respondents felt that the work environment poses a high risk for contracting the infection and did not feel safe using the standard infection-control measures. One reason for the observed low attitude score might have been the lack of HCW experience with exposure to such outbreaks. Due to the potential rapid dissemination of Covid-19 within the public and a large probability of a countrywide outbreak, along with the country’s experience in battling this similar coronavirus (MERS-CoV), the KSA was amongst the leading bodies in the world for its swift community action and hospital preparedness. This study aims to assess perception and attitude of HCWs in Saudi Arabia with regard to Covid-19, and to identify potential associated predictors.

## Methods

### Study design and setting

This is a cross sectional study of HCW working at the medical cities of the Saudi Ministry of National Guard Health Affairs (MNG-HA). MNG-HA provide healthcare services to national guard service members and their dependents through large medical cities located in the 3 most densely populated regions of KSA, namely the Central, Western and Eastern regions. All facilities have been Joint Commission International (JCI) accredited since 2006. During the COVID-19, and following the first reported case in KSA, MNG-HA has taken drastic infection control measures that included the reduction of elective surgeries, stopping in person outpatient services, and introducing ER workflow to minimize Covid-19 cases flow through the main ER.

### Subjects and sampling technique

The target population of the current study was all HCW employed by the MNG-HA at all three regions. An e-mail with an anonymous link to an electronic survey was sent to all HCWs who were on duty during the data collection period (~ 1400 HCWs), across all departments and specialties. This electronic survey was structured using the option that allowed for every participant to participate only once. The target sample size for the survey was estimated assuming a prevalence of high concern among HCW of 25.2% which was observed in another study in the same setting [16]. We estimated the sample needed for the survey to be 800 participants, assuming 95% confidence limits and 3% precision. Those who agreed to participate and who responded with completed questionnaires totaled 844 HCWs, with a response rate of 60.2%.

### Data collection

A structured, self-administered survey of HCWs was conducted via email, using a concern scale to assess their concern about Covid-19 pandemic. This survey was designed based on a validated concern scale previously used in a study of the concerns of HCWs with regard to MERS-CoV^16^. The scale consists of 32 statements that cover 5 domains; self-satisfaction, social status, work environment, infection control measures, government action and activities [16]. The scale was modified to also include a statement about the perception of HCWs towards curfew: “*I feel safe that government implemented the curfew and the movement restriction periods*”. A copy of the revised concern scale was attached as a supplementary material.

Data on gender, age, nationality, marital status, level of education, living status, professional characteristics and contact with patients were collected. HCWs were categorized according to their direct contact with Covid-19 patients to “Direct contact group”, or “Non Direct contact group”. The Direct contact group included all subjects caring directly for patients in the ER, Ward, or ICU. All statements were coded using 4 points Likert scale, taking values from 0 (“strongly disagree”) to 3 (“strongly agree”) resulting in a total concern score that ranges from 0 to 96. Participants were further classified into one of three groups based on their total concern score. The first group included subjects below the first quartile of the concern score (score of 39 and below), the second group included subjects with concern score between the 25th percentile (concern score of 40) and 75th percentile (concern score of 55) and the third group included subjects above the 75th percentile (score of 56 and above) [16].

The survey was distributed in the English language, as an electronic survey, to all HCWs via a link attached to a mass e-mail distribution, with no identifiers. A cover letter was attached to an email as a link sent to HCWs in their office emails, during the period between 15 and 30 of April, 2020. Study participants were expected to complete the survey and return it back without identifiers.

### Ethical issues

Participation in this study was voluntary. HCWs were assured in a written informed consent that their responses would remain anonymous and would not affect their performance evaluations, work status or compensations. HCWs were asked to respond to the survey if they agree on the informed consent. This study was approved by the institutional review board of the MNG-HA in Riyadh, Saudi Arabia (April 15, 2020; RC 20/173/R).

### Data analysis

All categorical variables including age, gender and occupation status were summarized and reported using frequency and proportions. The total concern score was summarized and reported using mean and standard deviation. Association of categorical variables with the different levels of concern was analyzed using the Chi square test for homogeneity. All continuous variables were compared across the different concern levels using the student-t test and one-way ANOVA. Multiple linear regression analysis was used to determine significant predictors of high concern scores to Covid-19 pandemic. For all statistical analyses, significance was considered at a *p* value of ≤0.05. All analyses were performed in the Statistical Package for the Social Sciences software (SPSS version 26.0; IBM Corporation, Armonk, NY, USA).

## Results

### Personal characteristics

A total of 844 MNG-HA HCWs responded to the survey (326 males and 518 females). They had an average age of 40.4 ± 9.5 years, 436 (51.7%) were from the central region, 183 (21.7%) from the eastern region and 225 (26.7%) from the western region. A total of 40.3% were nurses, 59.2% had direct patient contact, and 80.8% were living with family members and/or others, Table 1.

**Table 1:** Sociodemographic characteristics of HCWs at Ministry of National Guard-Health Affairs in different regions of Saudi Arabia

	Central Region no.(%)	Eastern Region no.(%)	Western Region no.(%)	Total no.(%)
**Total**	**436 (51.7)**	**183 (21.7)**	**225 (26.7)**	**844 (100.0)**
**Gender**				
Male	158 (36.2)	75 (41.0)	93 (41.3)	326 (38.6)
Female	278 (63.8)	108 (59.0)	132 (58.7)	518 (61.4)
χ2 = 2.17, df = 2, *p* = 0.34				
**Age (years)**				
≤ 30	76 (17.4)	18 (9.8)	12 (5.3)	106 (12.6)
30--45	253 (58.0)	103 (56.3)	142 (63.1)	498 (59.0)
> 45	107 (24.5)	62 (33.9)	71 (31.6)	240 (28.4)
x ± SD	38.9 ± 9.7	41.8 ± 8.9	42.0 ± 9.3	40.4 ± 9.5
χ2 = 24.62, df = 4, *p* < 0.001*				
**Marital Status**				
Single	182 (41.7)	42 (23.0)	59 (26.2)	283 (33.5)
Married	254 (58.3)	141 (77.0)	166 (73.8)	561 (66.5)
χ2 = 27.78, df = 2, *p* < 0.001*				
**Nationality**				
Saudi	180 (41.3)	66 (36.1)	94 (41.8)	340 (40.3)
Non Saudi	256 (58.7)	117 (63.9)	131 (58.2)	504 (59.7)
χ2 = 1.74, df = 2, *p* = 0.42				
**Education Level**				
Diploma	58 (13.3)	29 (15.8)	40 (17.8)	127 (15.0)
BS	284 (65.1)	103 (56.3)	120 (53.3)	507 (60.1)
MS/PHD	94 (21.6)	51 (27.9)	65 (28.9)	210 (24.9)
χ2 = 10.12, df = 4, *p* = 0.039				
**Job title**				
Physician/dentist/pharmacist	106 (24.3)	49 (26.8)	66 (29.3)	221 (26.2)
Nursing	189 (43.3)	57 (31.1)	94 (41.8)	340 (40.3)
Technician	47 (10.8)	36 (19.7)	23 (10.2)	106 (12.5)
Administrative	94 (21.6)	41 (22.4)	42 (18.7)	177 (21.0)
χ2 = 16.66, df = 6, *p* = 0.011*				
**Direct patient contact**				
Yes	248 (56.9)	121 (66.1)	131 (58.2)	500 (59.2)
No	188 (43.1)	62 (33.9)	94 (41.8)	344 (40.8)
χ2 = 4.69, df = 2, *p* = 0.10				
**Any family member/colleague/ friend tested positive for Covid-19**				
yes	24 (5.5)	3 (1.6)	17 (7.6)	44 (5.2)
No/Don’t know	412 (94.5)	180 (98.4)	208 (92.4)	800 (94.8)
χ2 = 7.30, df = 2, *p* = 0.026*				
**Status of living**				
Alone	78 (17.9)	33 (18.0)	51 (22.7)	162 (19.2)
With others	358 (82.1)	150 (82.0)	174 (77.3)	682 (80.8)
χ2 = 2.39, df = 2, *p* = 0.30				

*χ2* Pearson Chi-square test, *df* degree of freedom, *BS* Bachelor of Science, *MS* Master of Science, *PHD* Doctor of Philosophy

### Concerns of HCWs regarding Covid-19 pandemic

The majority of participants (72.1%) had an overall concern score of 55 or less out of a maximum score of 96 points. The responses to the 32 items in the questionnaire varied considerably. With regard to self-satisfaction domain, responses of concern varied from a high of 69.9% who expressed fear of getting infected from an infected colleague, to a low of 26.9% who felt unconfident a colleague would care for them if they contract the disease. In social status-related domain, concern varied from a high of 95.7% agreeing that they should limit their social activities due to Covid-19 to a low of 16.5% not feeling satisfied of telling their family if they get infected. In workplace-related domain, responses ranged from a high of 64.2% preferring to be absent from work to lower the chance of getting infected to a low of 6.6% agreeing they would feel ashamed telling their managers/colleagues if contracting Covid-19. In infection control-related domain, responses varied from a high of 71.6% not feeling there was a plan for Covid-19 outbreak in their area to a low of 22.3% did not feel an IC specialist is accessible to respond to their concerns and 27.7% did not feel safe at work when using the standard precautions. In the government-related domain, responses varied from a high of 94.7% agreeing with travel restrictions implemented by the government to a low of 23.7% agreeing that Covid-19 was not discussed efficiently in the media, Table 2.

**Table 2:** Responses of health-care workers to concern statements with the Covid-19 in Saudi Arabia

	Agree/ Strongly Agree *n* (%)		Disagree/ Strongly Disagree *n* (%)	
**A. Self-satisfaction domain**				
1. I feel unsafe working at my workplace.	406	48.1	438	51.9
2. I feel anxious while working with a febrile patient.	533	63.2	311	36.8
3. I feel at risk to contract a Covid-19 infection at work.	639	75.7	205	24.3
4. I feel obliged to care for a Covid-19 -infected patient.	519	61.5	325	38.5
5. I feel hopeless I might eventually get a Covid-19 at work.	388	46.0	456	54.0
6. I feel threatened if one of my colleagues contracted Covid-19 .	590	69.9	254	30.1
7. If I get Covid-19, I don’t feel confident an employee will care for me?	227	26.9	617	73.1
**B. Social status-related domain**				
1. I feel that I should limit my social activities due to Covid-19 .	808	95.7	36	4.3
2. I feel I will transmit Covid-19 to my family members.	492	58.3	352	41.7
3. I feel that my family members avoid me since I work in hospital.	219	25.9	625	74.1
4. I feel I should avoid leaving my home due to Covid-19 .	582	69.0	262	31.0
5. I feel my family will not look after me if I was infected.	142	16.8	702	83.2
6. I don’t feel confident telling my family and friends if I was infected.	139	16.5	705	83.5
**C. Workplace-related domain**				
1. I feel that my institution didn’t support me during the Covid-19 crisis.	164	19.4	680	80.6
2. I feel that my institution is losing control of the Covid-19 crisis.	103	12.2	741	87.8
3. I feel overwhelmed with the new Covid-19 regulations.	459	54.4	385	45.6
4. I feel Covid-19 crisis increased my workload.	338	40.0	506	60.0
5. I feel that the increase in workload was not meet with proper staffing.	333	39.5	511	60.5
6. I feel absence from work reduces the chance of getting Covid-19 .	542	64.2	302	35.8
7. In case I had Covid-19, I feel ashamed telling my manager/colleagues.	56	6.6	788	93.4
8. I feel I should change my current job due to Covid-19 crisis.	77	9.1	767	90.9
**D. Infection control-related domain**				
1. I am not confident with the current infection control measures.	243	28.8	601	71.2
2. I don’t feel proper infection control training has been offered to me.	229	27.1	615	72.9
3. I don’t feel an infection specialist is accessible to respond to my concerns.	188	22.3	656	77.7
4. I don’t feel there is Covid-19 outbreak plan set at my area.	604	71.6	240	28.4
5. I don’t feel safe at work when I use the standard precautions.	234	27.7	610	72.3
**E. Government-related domain**				
1. I feel the government should restrict travel from /to areas of disease.	799	94.7	45	5.3
2. I feel the government should isolate Covid-19 cases in special hospitals	784	92.9	60	7.1
3. I feel government should avoid inviting expatriates from infected areas.	640	75.8	204	24.2
4. I feel schools and shopping markets need to be closed to control Covid-19.	717	85.0	127	15.0
5. I don’t feel Covid-19 has been highlighted and discussed efficiently in media.	200	23.7	644	76.3
6. I feel safe that government implemented the curfew and the movement restriction periods.	790	93.6	54	6.4

*Abbreviation*: *Covid-19* coronavirus disease 2019

Overall, 27.9% of HCWs had high concern, 46.9% moderate concern and 25.2% low concern. The average concern score was 48.5 ± 12.8, out of a maximum possible concern score of 96. Level of concern was significantly associated with age (χ^2^ = 19.52; *p* = 0.001), marital status (χ2 = 6.30; *p* = 0.043), nationality (χ^2^ = 18.86; *p* < 0.001), level of education (χ^2^ = 13.48; *p* = 0.009), occupation (χ^2^ = 14.54; *p* < 0.001), geographical region of employment (χ^2^ = 11.09; *p* = 0.026), direct patient contact (χ^2^ = 6.88, *p* = 0.032) and status of living (χ2 = 14.54, *p* = 0.001), Table 3. In multiple regression analysis (Table 4), predictors of high concern scores were; HCWs of younger age (*p* = 0.003), Saudi nationality (*p* < 0.001), undergraduate education (*p* = 0.044), and those working in the western region (*p* = 0.003), living with others (*p* = 0.003) and in direct contact with patients (*p* = 0.018).

**Table 3:** Comparison between the levels of concern about Covid-19 and personal characteristics of healthcare workers in Saudi Arabia

Characteristics	Low concern (score = 0–39)	Moderate concern (score = 40–55)	High concern (score = 56–96)	Mean concern score
*Total*	213 (25.2)	396 (46.9)	235 (27.9)	48.5 ± 12.8
*Gender*				
Male	83 (25.5)	160 (49.1)	83 (25.5)	47.3 ± 12.2
Female	130 (25.1)	236 (45.6)	152 (29.3)	49.2 ± 13.1
	χ2 = 1.62, df = 2, *p* = 0.44			t = 2.14, *p* = 0.33
*Age (years)*				
≤ 30	19 (17.9)	47 (44.3)	40 (37.7)	50.4 ± 13.1
30–45	117 (23.5)	231 (46.4)	150 (30.1)	49.7 ± 13.1
> 45	77 (32.1)	118 (49.2)	45 (18.8)	45.2 ± 11.3
	χ2 = 19.52, ==0.001*			f = 11.60, *p* < 0.001*
*Marital status*				
Unmarried	61 (21.6)	129 (45.6)	93 (32.9)	49.5 ± 12.9
Married	152 (27.1)	267 (47.6)	142 (25.3)	48.0 ± 12.7
	χ2 = 6.30, df = 2, *p* = 0.043*			t = 1.58, *p* = 0.11
*Nationality*				
Saudi	72 (21.2)	146 (42.9)	122 (35.9)	51.1 ± 13.7
Non-Saudi	141 (28.0)	250 (49.6)	113 (22.4)	46.7 ± 11.8
	χ2 = 18.86, df = 2, *p* < 0.001*			t = 4.81, *p* < 0.001*
*Level of education*				
Diploma	38 (29.9)	54 (42.5)	35 (27.6)	48.0 ± 14.3
BS	107 (21.1)	246 (48.5)	154 (30.4)	49.6 ± 12.4
MSN/PHD	68 (32.4)	96 (45.7)	46 (21.9)	46.0 ± 12.4
	χ2 = 13.48, df = 4, *p* = 0.009*			f = 6.26, *p* = 0.002*
*Job title*				
Physician/Dentist/Pharmacist	59 (26.7)	105 (47.5)	57 (25.8)	46.9 ± 10.9
Nurse	75 (22.1)	168 (49.4)	97 (28.5)	49.4 ± 12.3
Technician	35 (33.0)	46 (43.4)	25 (23.6)	46.7 ± 12.6
Administrative	44 (24.9)	77 (43.5)	56 (31.6)	49.9 ± 15.5
	χ2 = 14.54, df = 6, *p* < 0.001*			f = 3.18, *p* = 0.023*
Geographical region of employment				
Central	124 (28.4)	192 (44.0)	120 (27.5)	47.9 ± 13.5
Eastern	50 (27.3)	88 (48.1)	45 (24.6)	47.8 ± 12.6
Western	39 (17.3)	116 (51.6)	70 (31.1)	50.1 ± 11.2
	χ2 = 11.09, df = 4, *p* = 0.026*			f = 2.2.58, *p* = 0.076
Direct patient contact				
Yes	120 (24.0)	224 (44.8)	156 (31.2)	49.1 ± 12.5
No	93 (27.0)	172 (50.0)	79 (23.0)	47.7 ± 13.2
	χ2 = 6.88, df = 2, *p* = 0.032*			t = 1.58, *p* = 0.11
Positive family member				
yes	11 (25.0)	21 (47.7)	12 (27.3)	49.4 ± 12.6
No/Don’t know	202 (25.3)	375 (46.9)	223 (27.9)	48.4 ± 12.8
	χ2 = 0.013, df = 2, *p* = 0.99			t = 0.48, *p* = 0.63
Living condition				
Alone	59 (36.4)	70 (43.2)	33 (20.4)	45.1 ± 12.8
With others	154 (22.6)	326 (47.8)	202 (29.6)	49.3 ± 12.7
	χ2 = 14.54, df = 2, *p* = 0.001*			t = 3.84, *p* < 0.001*

*χ2* Pearson Chi squared test, *f* Analysis of variance (ANOVA) test, *--Statistically significant difference, *df* degree of freedom

**Table 4:** Multiple regression analysis of concern scores about Covid-19 among healthcare workers in Saudi Arabia

Independent variables	β	*SE*	t-value	*p*-value.
Males* versus females	−1.381	.982	−1.406	.160
Age (in years)	−.154	.051	−3.029	.003*
Married* versus unmarried	.009	1.012	.009	.993
Saudi* versus non-Saudi	3.825	.977	3.916	<.001*
Diploma versus higher* education	−2.373	1.176	−2.017	.044*
Physicians* versus others	−1.858	1.268	−1.466	.143
Western* versus other regions of employment	2.931	.968	3.029	.003*
Living with others* versus living alone	3.410	1.153	2.957	.003*
A family member/colleague/friend tested positive* versus negative, for Covid-19	.472	1.915	.246	.806
Direct* versus indirect contact with patients	2.097	.882	2.378	.018*
(Constant)	49.914	2.388	20.902	.000

*β* beta coefficient, *SE* standard error, *t* t statistics, *---reference category, **---significant association

## Discussion

This study aimed to assess perception and attitude of HCWs in Saudi Arabia with regard to Covid-19, and to identify potential associated predictors. An overall average concern score of 48.5 ± 12.8 out of a maximum possible score of 96 points was observed, with a negative range of attitude, indicating a moderate level of concern. In comparison with the results of a previous survey in the same settings using the same data collection tool, to assess the concern of HCWs about MERS outbreak in Saudi Arabia [16], HCWs reported significantly higher mean concern scores about Covid-19 pandemic. This may reflect the impact and role of mass media and social media marketing on the way we perceive our world and our everyday lives on individual, social and societal levels, during these critical times. Even with the help of the media, this pandemic has had worldwide repercussions and is not yet controlled in some countries. A study was carried out on 582 HCWs at King Khalid University Hospital (KKUH), Riyadh, Saudi Arabia, showed that the majority of HCWs had mild anxiety from Covid-19 [13]. However; the survey was conducted before registering any case of Covid-19 in Saudi Arabia.

An important finding in the present study was that a high level of concern about Covid-19 pandemic was prevalent across the different concern domains. The highest level of concern was observed in the HCWs’ responses to questions regarding fears of infection of a family member, fears of being in public places that may result in infection, the closure of schools and workplaces in the event of an epidemic and risks associated with dealing with a febrile patient, obligation of care provision for patients infected with Covid-19 and government’s action to implement the curfew and the movement restriction periods. It was interesting that in the present study, 85% agreed that school and shopping markets need to be closed, while only 19% during the previous MERS outbreak [16]. This finding may reflect the perception of HCWs in our study of the magnitude of Covid-19 pandemic. However, it is important to note that this perception of fear might differ from country to another. For example in Japan with the absence for an epidemic during the SARS-COV outbreak, more than 50% reported having a high level of fear and an anxiety of infection [19], while in Thai study, nearly all HCWs reported acceptance to take the risk of caring for H5N1 patients [20].

In line with the WHO recommendations for institutional preparedness to reduce the impact of potential outbreaks, MNG-HA has developed a comprehensive plan of medical and public health response for Covid-19 epidemic [21]. This plan aimed at the protection of HCWs through the implementation of strict infection control measures and personal protection practices. Despite these efforts, HCWs in our study did not feel safe at the workplace and felt at risk of contracting the infection. This finding is similar to a study in the UK in which 66% of the HCWs did not feel confident in the healthcare system’s ability to cope with bird flu epidemic [22].. The exact reasons of such high concern among HCWs, despite the existence of a preparedness plan, cannot be determined from the current study and further studies are needed.

Our study shows that HCWs who were in direct contact with patients had significantly higher concern scores than those who were not in direct contact. This finding was in agreement with the results of a study in China [23] to compare the average values of fear, anxiety and depression due to Covid-19 pandemic between medical and admin staff, where medical staff reported greater fear, anxiety and depression than administrative staff. This finding is not surprising given the higher perceived risk by those HCW due to the condition of the work environment. However it is important to pay special attention to those HCWs to manage their perception of risk by ensuring that they have access to proper personal protective equipment (PPE) and safe patients’ handling procedures [24].

Saudi HCWs, in the present study, reported higher concern to Covid-19 pandemic as compared to non-Saudis. This can be explained by the culture norms and the difference in living conditions between Saudis and non Saudi HCW. The majority of non saudi HCW are expats who are likely to live alone with their family memebrs living in their home countries. Therfore expats are less likely to worry about the risk of infecting their family members and loved one compared to Saudi HCW who live with their families and tend to have a very active social life [25].. The present study also showed that living with others was an independent predictor of high level of concern about Covid-19 infection, most likely due to their fear of transmitting the infection to others if they get infected.

An interesting but a little counterintuitive finding of our study is the fact that older HCWs were less concerned about covid-19 than the younger ones. This is especially true given that risk factors for severe disease and death in Covid-19 include older age among many other factors [26]. However, this finding could be attributed to the fact that oldest HCW’s could not be working in direct contact to patients, due to the higher risk of severe disease. Further, there was a significant association between higher concern score and lower education level. In a survey on the undergraduate medical students in 3 medical institutes of Karachi, the majority of students found worrisome of getting infected with Covid-19 during medical rotations, dreaded insufficient care and inappropriate treatment if they acquire infection and thought their institute-associated hospital won’t be able to handle the situation in case of an uncontrolled outbreak [27]. One possible explanation can be inferred from the theory of reasoned action of a causal relationship between knowledge and experience and the subsequent positive perception and intention to change behavior [28]attitudes and behavioral intent.

In the current study HCWs of western region had significantly higher concern score compared to other regions. This was different than the study during MERS where the HCWs of central region had higher concern than other regions [16]. We believe that these differences are likely due to the perception of HCWs of the magnitude of the pandemic in the different regions. During Covid-19, the western region had shown much rapid increase of confirmed cases compared to the other regions [6]. Additionally, the government has implemented complete lockdown of the western region prior to other regions. However during MERS, the largest outbreak has taken place in the central region. The large magnitude of the epidemic the western region compared to other regions in the country could have contributed to the observed level of concern of HCWs in this region.

### Limitations

Our study is not without limitation. Our survey was based on self-reported information which might suffer from a recall bias. Moreover, all study participants were HCWs in tertiary hospitals, and therefore could limit the generalizability of the findings to other settings. Finally, all identified predictors of concerns cannot be interpreted beyond general association. Despite these limitations, our study addresses a major problem faced by HCWs in many countries around the world during this pandemic.

## Conclusions

The current study highlights the high concern among healthcare workers about Covid-19 and identifies the predictors of those with the highest level of concern. High level of concern could lead to suboptimal healthcare service as well as less effective management of COVID-19 cases. This could be mitigated by implementing strategies designed to minimize perceived risk of infection by HCWs. These strategies should be part of the early planning for a response to an epidemic and it should cover a wide range of programs that focus on financial incentives, education, personal counseling and education.

## Supplementary information

**Additional file 1.**

## Data Availability

Most of the data supporting our findings is contained within the manuscript, and all others, excluding identifying/confidential patient data should, will be shared upon request.

## Abbreviations

KAMC: King Abdulaziz Medical city
MNG-HA: Ministry of National Guard-Health Affairs
Covid-19: Coronavirus disease 2019
SARS-CoV-2: Severe Acute Respiratory Syndrome Coronavirus-2
MERS-CoV: Middle East Respiratory Syndrome-corona virus
HCWs: Health care workers
WHO: World Health Organization
PHEIC: Public Health Emergency of International Concern
IHR: International Health Regulation
CFR: Case fatality rate
ER: Emergency department
ICU: Intensive care unit
IRB: Institutional Review Board
