# Supplementary material for: Perception and attitude of healthcare workers in Saudi Arabia with regard to Covid-19 pandemic and potential associated predictors
Source: BMC Infect Dis. 2020 Sep 29;20:719. doi: 10.1186/s12879-020-05443-3 (PMC7523489; doi:10.1186/s12879-020-05443-3)
Supplement: Supplementary file 1 — Additional file 1. [file 12879_2020_5443_MOESM1_ESM.pdf]

RYD-20-419812-53422

King Abdullah International Medical Research Center
KAIMRC
APPROVED
IRB
Institutional Review Board

Ministry of NATIONAL GURAD HEALTH AFFAIRS

KING ABDULLAH INTERNATIONAL MEDICAL RESEARCH CENTER

Dear MNG-HA employee,

In December 2019, a cluster of patients with pneumonia was linked to a seafood wholesale market in Wuhan, China, which lead to the discovery of a new betacoronavirus,1 on 7 January, 2020, named Severe Acute Respiratory Syndrome Coronavirus-2 (SARS-CoV-2) that causes coronavirus disease 2019 (COVID-19). With its novelty and rapid national and international spread on 30 Jan 2020, the World Health Organization (WHO) International Health Regulation (IHR) emergency committee declared the disease a Public Health Emergency of International Concern (PHEIC). It was declared as a worldwide pandemic3 on 11 March 2020.

In order to better understand Covid-19 outbreak from your perspective, KAIMRC launched a study to assess your concerns, and attitudes towards various aspects such as personal safety, job-related, social status, and governmental regulations. The survey is voluntary and anonymous, so you won't be identified by your name or badge number. However, your participation is highly recommended as your input will provide us with better understanding of the current issue.

For additional information about this study, please contact:

Professor Mostafa A. Abolfotouh

King Abdullah International Medical Center

Tel: 00966 1142 94460

Concern toward Covid-19 outbreak among hospital-based health-care workers of Ministry of National Guard, Saudi Arabia

I. Please fill in the answers that best describe you:

1	Gender:	O Female O Male
2	Age:	……………(yrs)
3	Marital Status:	O Unmarried O Married
4	Nationality	O Saudi O Non-Saudi
5	Level of Education	O BS O Diploma<br>O MSN/PHD
6	Job title:	O Physician O Nursing<br>O Technician O Others:…………
7	Housing status	O Alone O With Family<br>O With Others
8	Geographical region of employment	O East O Central O West
9	Do you have any of your family members/colleagues/friends who tested positive for COVID-19?	O Yes O No O Do not know
10	Are you in direct contact with patients	O Yes O No

II. Please fill in the answers that best describes your personal feelings:

**A. Self satisfaction domain**	Strongly Agree	Agree	Disagree	Strongly Disagree
1. I feel unsafe working at my work place				
2. I feel anxious while working with a febrile patient				
3. I feel at risk to contract a Covid-19 infection at work				
4. I feel obliged to care for a Covid-19 infected patient				
5. I feel hopeless I might eventually get a Covid-19 at work				
6. I feel threatened if one of my colleagues contracted Covid-19				
7. If I get Covid-19, I don’t feel confident an employee will care for me?				

**B. Social status related domain**	Strongly Agree	Agree	Disagree	Strongly Disagree
1. I feel that I should limit my social activities due to Covid-19.				
2. I feel I will transmit Covid-19 to my family members.				
3. I feel that my family members are avoiding me since I work in hospital				
4. I feel I should avoid leaving my home due to Covid-19				
5. I feel my family will not look after me if I was infected.				
6. I don't feel confident telling my family and friends if I was infected				
**C. Work place related domain**	Strongly Agree	Agree	Disagree	Strongly Disagree
1. I feel that my institution didn't support me during the Covid-19 crisis.				
2. I feel that my institution is loosing control of the Covid-19 crisis				
3. I feel overwhelmed with the new Covid-19 regulations				
4. I feel Covid-19 crisis increased my workload.				
5. I feel that the increase in workload was not met with increase staffing				
6. I feel absence from work reduces the chance of getting Covid-19.				
7. In case I contracted Covid-19, I feel ashamed telling my manager and work colleagues?				
8. I feel I should change my current job due to Covid-19 crisis				

**D. Infection control related domain**	Strongly Agree	Agree	Disagree	Strongly Disagree
1. I am not confident with the current infection control measures				
2. I do not feel proper infection control training has been offered to me				
3. I do not feel an infection control specialist is accessible to respond to my concerns				
4. I do not feel safe at work when I use standard precautions				
5. I feel there is Covid-19 outbreak plan set at my area.				
6. I feel safe at work when I use the standard precautions				

**E. Government related domain**	Strongly Agree	Agree	Disagree	Strongly Disagree
1. I feel the government should restrict travel from and to the areas of disease				
2. I feel the government should isolate Covid-19 cases in special hospitals.				
3. I feel the government should avoid inviting expatriates from infected areas.				
4. I feel schools and shopping markets need to be closed to control Covid-19 crisis.				
5. I feel safe that government implemented the curfew and the movement restriction periods.				
6. I do not feel Covid-19 has been highlighted and discussed efficiently in media.				
6. Depressions status:<br>All in all, I feel depressed due to the Covid-19 crisis.				

Best Regards
